# An intervention delivered by text message to increase the acceptability of effective contraception among young women in Palestine: study protocol for a randomised controlled trial

**DOI:** 10.1186/s13063-017-2191-1

**Published:** 2017-10-03

**Authors:** Ona L McCarthy, Ola Wazwaz, Iman Jado, Baptiste Leurent, Phil Edwards, Samia Adada, Amina Stavridis, Caroline Free

**Affiliations:** 1 Department of Population Health, Faculty of Epidemiology and Population Health, London School of Hygiene and Tropical Medicine, Keppel Street, London, WC1E 7HT UK; 2 Palestinian Family Planning and Protection Association, Industrial Zone, Wadi Al-Joze, Jerusalem, Palestine; 3 Department of Medical Statistics, Faculty of Epidemiology and Population Health, London School of Hygiene and Tropical Medicine, Keppel Street, London, WC1E 7HT UK; 4 International Planned Parenthood Federation, Arab World Regional Office, 2 Place Virgile, Notre Dame, Tunis 1082 Tunisia

**Keywords:** Palestine. Contraception, Mobile phone, Cell phone, Reproductive health, Young adults

## Abstract

**Background:**

Unintended pregnancy can negatively impact women’s lives and is associated with poorer health outcomes for women and children. Many women, particularly in low- and middle-income countries, continue to face obstacles in avoiding unintended pregnancy. In the State of Palestine, a survey conducted in 2006 estimated that 38% of pregnancies are unintended. In 2014, unmet need for contraception was highest among young women aged 20–24 years, at 15%.

Mobile phones are increasingly being used to deliver health support. Once developed, interventions delivered by mobile phone are often cheaper to deliver than face-to-face support. The London School of Hygiene and Tropical Medicine and the Palestinian Family Planning and Protection Association have partnered to develop and evaluate a contraceptive behavioural intervention for young women in Palestine delivered by mobile phone. The intervention was developed guided by behavioural science and consists of short, mobile phone text messages that contain information about contraception and behaviour change methods delivered over 4 months.

**Methods:**

We will evaluate the intervention by conducting a randomised controlled trial. Five hundred and seventy women aged 18–24 years, who do not report using an effective method of contraception, will be allocated with a 1:1 ratio to receive the intervention text messages or control text messages about trial participation. The primary outcome is self-reported acceptability of at least one method of effective contraception at 4 months. Secondary outcomes include the use of effective contraception, acceptability of individual methods, discontinuation, service uptake, unintended pregnancy and abortion. Process outcomes include knowledge, perceived norms, personal agency and intervention dose received. Outcomes at 4 months will be compared between arms using logistic regression.

**Discussion:**

This trial will determine the effect of the intervention on young women’s attitudes towards the most effective methods of contraception. If the intervention is found to be effective, the intervention will be implemented widely across Palestine. The results could also be used to design a larger trial to establish its effect on unintended pregnancy.

**Trial registration:**

ClinicalTrials.gov, ID: NCT02905461. Registered on 14 September 2016.

**Electronic supplementary material:**

The online version of this article (doi:10.1186/s13063-017-2191-1) contains supplementary material, which is available to authorized users.

## Background

In 2012, an estimated 85 million pregnancies worldwide were unintended, half of which ended in abortion [1]. Women who have unintended pregnancies can experience decreased psychological wellbeing [2–10], initiate antenatal care later than those with intended pregnancies [4, 10–14] and access care less frequently [4, 11, 14]. There is a higher risk of low birth weight and pre-term birth among children born of unintended pregnancies [15, 16] and these children can exhibit behavioural problems more often than children born of intended pregnancies [17]. Unintended pregnancy can also impact the social wellbeing of parents and families. It can delay or prevent educational and career achievements, which can impact the financial security of the family [18]. Unsafe abortions are a consequence of unintended pregnancy where access to safe abortion is limited [19, 20]. Satisfying unmet need for modern contraception reduces unintended pregnancies and identifying the barriers to non-use is crucial in achieving this [21, 22].

The conflict in the State of Palestine (the West Bank, East Jerusalem and the Gaza Strip, hereafter referred to as ‘Palestine’) has negatively impacted the health and wellbeing of Palestinians [23–25]. The conflict has also had negative effects on reproductive health in Palestine [26, 27]. Data regarding unintended pregnancy in Palestine generally comes from household surveys where only married women (sexual activity before marriage is stigmatised in Palestinian culture) are asked if their current or last pregnancy was intended at the time that they became pregnant [28]. Effective contraception methods are those with a less than 10% typical use-failure rate at 12 months [29–31]. The (non-permanent) effective methods available in Palestine are oral contraceptive pills (OCs), intrauterine devices (IUDs), injectables, implants, and the patch. Despite the availability of these methods, a 2006 survey estimated that 38% of pregnancies in Palestine are unintended [28, 32].

In 2014, the unmet need for contraception was highest among young women aged 20–24 years, at 15% [33]. The modern contraceptive prevalence rate among married women aged 15–24 years is estimated to be 24% and the effective rate in the same group is estimated to be 17% [33]. Barriers to contraceptive uptake are lack of accurate and comprehensive information about a range of contraceptive methods, lack of spousal communication regarding contraception, peers’ and relatives’ (particularly husband and mother-in-law) disapproval of contraception, societal pressure to bear children early in marriage and inadequate family planning services [34–38]. Attitudes, such as perceived inconvenience and fear of the side effects of contraceptive methods along with husbands’ opposition, are common reasons married women provide for not using contraception [39, 40]. Education also is a factor in this setting as Palestinian women who spend more time in education report fewer unintended pregnancies [28]. A non-representative study in 2014 and found that 55% of women aged 15–49 years from a community sample (from underserved areas) said that their pregnancy was unintended (‘unwanted’) and, of these, 26% said that this was because it ‘was not their choice’. In a client sample (from service-delivery points), 40% reported unintended pregnancy, with 32% saying that it ‘was not their choice’ [41].

Mobile phones are increasingly being used to deliver health support over a range of health behaviours [42–52]. In sensitive areas, such as reproductive and sexual health, short messages delivered by mobile phone may be advantageous as they have the potential to be read at a time and place of the recipient’s choosing. The support can be non-judgemental and is often more convenient and cheaper to deliver than face-to-face support. In Palestine, where there is a substantial area that is underserved with regard to sexual and reproductive health services [41], delivering contraceptive support by mobile phone may be a particularly advantageous mode by which to reach people. Those that do have access to services may find the barriers outlined above difficult to overcome and may also benefit from mobile phone support. While there is some evidence from high-income countries that mobile phone-based interventions can increase contraceptive-related behaviours [53–55] and knowledge [56], none of the trials evaluating these interventions had a low risk of bias [57]. To the best of our knowledge, there is only one trial conducted in a non-high-income country (Cambodia); this trial found that post-abortion voice messaging with telephone counselling support increased effective contraceptive use [58].

A systematic approach to developing behaviour change interventions is recommended [59–61] as it allows for the intervention to be clearly defined. The London School of Hygiene and Tropical Medicine (LSHTM) and the Palestinian Family Planning and Protection Association (PFPPA), a Member Association of the International Planned Parenthood Federation (IPPF), are collaborating to evaluate a contraceptive behavioural intervention delivered by mobile phone for young women in Palestine. We developed the intervention guided by an established approach based on behavioural science [62]. Among other activities, the approach involved consultation with young people, which explored their knowledge of, attitudes towards, and barriers in, using contraception; specifying behavioural change; identifying behaviour change methods and producing the intervention content through an interactive process of writing, testing with young people and refining.

In Palestine, mobile phone ownership is high, with 92% of all adults owning a mobile phone and among people aged 18–34 years, 73% report owning a smartphone [63]. However, the intervention development process revealed that many young people in Palestine did not have regular Internet access on their mobile phones. Those who sometimes access the Internet though their mobile data said that it is common for the connection to be lost. Because of this and because many of the young people we consulted with preferred it, we identified short messaging service (SMS) as the most appropriate mode of intervention delivery.

This randomised controlled trial will evaluate the effect of the intervention on young women’s attitudes towards the (non-permanent) effective contraceptive methods available in Palestine: OCs, IUDs, the injection, the implant, and the patch. Sexual activity before marriage is highly stigmatised in Palestine and there is strong cultural pressure to bear children soon after marriage. While it is estimated that 24% of women are married before age 18 years [33], a significant proportion of participants in the study population will likely be either not married (and not sexually active or not willing to admit that they are) or newly married. Because of this, an objective primary outcome of effective contraceptive use would not be advisable as powering a study for an outcome with a small number of events would make the sample size prohibitively large. In not-married/not-sexually-active young women, the intervention aims to increase acceptability of the effective methods for when they may want to limit or space their families and could benefit from finding a range of methods acceptable.

This will be the first trial evaluating an intervention delivered by mobile phone that is designed to increase the acceptability of effective contraception in Palestine [57]. The results of the study will contribute to the growing body of research on the utility of mobile phones as an intervention-delivery mechanism and contribute to the evidence base for contraceptive interventions for young women in Palestine.

## Methods

### Study design

This study is a parallel-group, individually randomised superiority trial with a 1:1 allocation ratio evaluating the effect of a contraceptive intervention delivered by mobile phone text messaging compared with control text messages about trial participation. The objective of this research is to establish the effect of the intervention on young women’s attitudes towards effective contraception in Palestine.

### Eligibility criteria

Women aged 18–24 years, who do not report using effective contraception, own a personal mobile phone, live in the West Bank, can provide informed consent and can read Arabic will be eligible to take part. The lower age limit of 18 years was chosen because it is the age in Palestine where people are able to provide independent informed consent to take part in research. The upper age limit of 24 years was chosen because this most closely matched the target group ‘young people’ [64] which was identified by the funder. Participants must also be willing to receive messages about contraception on their mobile phone.

### Recruitment

The trial will be promoted through PFPPA’s service-delivery points through outreach sites, the PFPPA website, the distribution of trial promotional material via flyers and social media sites. PFPPA service-delivery points provide: contraceptive methods; counselling for women in psychological, legal and social matters; laboratory tests for both men and women; maternal, antenatal and post-natal care and infertility services [65]. The promotional material includes brief information about the trial (e.g. who is conducting it, who may be eligible, what participation would involve) with a link to the secure trial database and randomisation system.

To maximize the chance of recruiting to target, LSHTM conducted a pre-trial training in Bethlehem to train local staff on all recruitment procedures. The training included discussions about the practicalities of recruitment with a view to developing the most appropriate strategies.

**Fig. 1:**
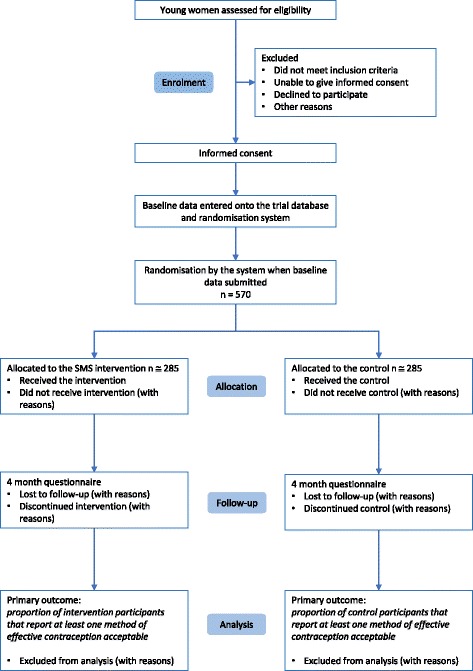
Consolidated Standards of Reporting Trials (CONSORT) diagram

We will report the number of people assessed for eligibility, excluded before randomisation, the number of participants randomised, allocated to the intervention, completed follow-up and analysed (Fig. 1. Consolidated Standards of Reporting Trials (CONSORT) diagram).

### Intervention

The intervention is informed by the Integrated Behavioural Model [66] and consists of short mobile phone text messages providing contraceptive support delivered over 4 months. The intervention messages provide information about contraception, target beliefs identified in the development phase that influence contraceptive use (e.g. misconceptions about the side effects and health risks of contraception, belief that non-hormonal methods are better because they are not harmful to health) and aim to support young women in believing that they can influence their reproductive health. The intervention provides accurate information about contraception and contains the following behaviour change methods, adapted for delivery by mobile phone [67]: belief selection, facilitation, anticipated regret, guided practice, verbal persuasion, tailoring, cultural similarity, arguments, shifting perspective and goal setting.

The messages are tailored according to marital status, resulting in two sets of intervention messages: (1) female-married and (2) female-not married. Most of the messages in the two sets overlap, with minor tailoring so that the messages are relevant to marital status (a proxy for sexual activity). Participants allocated to the intervention group receive zero to three messages per day (113 messages for female-not married and 120 messages for female-married) for 120 days.

The message sets start with 6 days of messages (10 messages for married women and 11 for not-married women) with general information about the study, such as information about what they will receive over the next 120 days, how to stop the messages, who to contact if they change their number, how to keep the messages private and information about who to call if they feel unsafe as a result of someone reading the messages. Included in the intervention messages that the intervention recipients receive are seven control messages about the importance of their participation and reminding them to contact the project coordinator if they change their number. On days 119 and 120, the message sets include four messages that indicate that the messages have ended, provide information on how to complete the follow-up questionnaire, reassurance that the information that they provide is confidential and a final message stating that their participation is helping to determine the best ways to provide reproductive health services in Palestine.

Details regarding the development of the intervention and intervention description will be reported in a forthcoming publication.

### Control

Participants allocated to the control group receive 16 messages over 120 days. The first 4 days include six messages that introduce the study, provide information about what they will receive over the next 120 days, how to stop the messages and who to contact if they change their number (e.g. ‘Thank you for joining. This study is being conducted by Palestinian Family Planning and Protection Association’; ‘Over the next 4 months, we will send you a few messages a month’). They will then receive two messages a month for 3 months – one about the importance of their participation and one reminding them to contact the project coordinator if they change their number (e.g. ‘Your participation in the study is a way to actively be involved in matters that affect your life. Thanks’; ‘If you change your number, please contact X to let us know’). On day 105, participants will receive one message about the importance of their participation (‘Your participation helps in reducing inequalities in reproductive health for youth’). On day 120, they will receive three messages that provide information on how to complete the follow-up questionnaire, reassurance that the information that they provide is confidential and a final message stating that their participation is helping to determine the best ways to provide reproductive health services in Palestine (e.g. ‘It is time to complete the final questionnaire. Research staff will call you in few days or you can complete it here LINK’; ‘You are helping us determine the best ways to provide reproductive health services in Palestine’).

All participants will receive usual care and will be free to seek any other support, whether existing or new.

### Outcomes

#### Primary outcome

The primary outcome is the proportion of participants reporting that at least one method of effective contraception is acceptable at four months post randomisation. The acceptability of each method is binary (acceptable/not acceptable), but is derived from ordinal data from the following stems: Using the [method]… causes infertility, …causes unwanted side effects, …is easy, …is a good way to prevent pregnancy and I would recommend the [method] to a friend. The IUD and implant include an additional stem: The [method] insertion would not be a problem for me. The response options for each scale are: strongly disagree, disagree, not sure, agree, strongly agree and I do not know what the [method] is. A method is acceptable if participants report ‘agree’ or ‘strongly agree’ for all scales except for ‘…causes infertility’ and ‘…causes unwanted side effects’ stems, for which ‘disagree’ or ‘strongly disagree’ denotes acceptability (items 1-27 in Additional file 1 and items 4-30 in Additional file 2).

#### Secondary outcomes

Secondary outcomes are: the proportion reporting current use of effective contraception (use of effective contraception); the proportion reporting that each effective contraceptive method is acceptable (acceptability of individual methods); the proportion reporting use of effective contraception at any time during the four months (discontinuation); the proportion reporting attending a sexual health service during the four months (service uptake); the proportion reporting that they became pregnant and did not want to become pregnant during the study (unintended pregnancy); the proportion reporting having an abortion during the study (induced abortion).

##### Process outcomes

The process outcomes are: knowledge of effective contraception; perceived norms and personal agency in relation to using and communicating with partners about contraception; intention to use effective contraception and intervention dose received.

### Data collection

Data will be collected at baseline and at 4 months post randomisation using questionnaires, which we tested for face validity with the target group. We asked people to comment on the length of the questionnaires, the comprehensibility of the questions, the meaning of the scales and suggestions for improvement. All data will be entered onto the trial database and randomisation system, which is on LSHTM’s secure server. At both time points, participants can either fill out a paper-based version of the questionnaire at the recruitment site, provide the data over the phone with research staff or enter data directly onto the online system, according to their preference. If participants provide their questionnaire data by paper or over the phone, research staff will enter this data onto the system.

### Baseline data collected

At baseline we will measure the primary outcome and collect the following personal and demographic data: full name; mobile phone number; email address; date of birth; marital status; number of children; residence; occupation; education level; current pregnancy intention; current method; how they heard about and enrolled in the study and the time that they prefer to receive the messages (see Additional file 1).

### Follow-up data collected

At 4 months, we will measure the primary, secondary and process outcomes and collect the following data: if participants report using an effective method, where they obtained it; current pregnancy intention; whether they knew someone else that took part in the study and, if so, if they read each other’s messages (contamination); if they have experienced physical violence since being in the study and if anything good or bad happened as a result of receiving the messages (see Additional file 2). Staff unaware of partcipants’ allocation will contact participants by phone to collect the follow-up data (participants can also complete the questionnaire online or attend the service). For participants who report use of effective contraception at follow-up, local research staff will attempt to locate the service records to objectively verify use.

### Methods to improve the quality of data collection

Closer to the start of follow-up, we will conduct a ‘follow-up refresher’ with staff who will collect follow-up data over the phone. This training will re-emphasise how to collect the follow-up data in a neutral, standardised way. Staff will also be given a suggested script and guidelines to follow when gathering the data.

### Methods to maximize follow-up response

The pre-trial training also included training in follow-up procedures. It emphasised the importance of ensuring that participants understand that participation involves completing a 4-month questionnaire and to potentially receiving daily messages about contraception for 4 months. The control messages, also sent to participants allocated to the intervention, are an effort to keep participants engaged. Staff will contact non-responders up to three times for their follow-up data. Follow-up will end 6 months after the last participant has been randomised or after staff have attempted to contact all non-responders three times, whichever comes first.

**Fig. 2:**
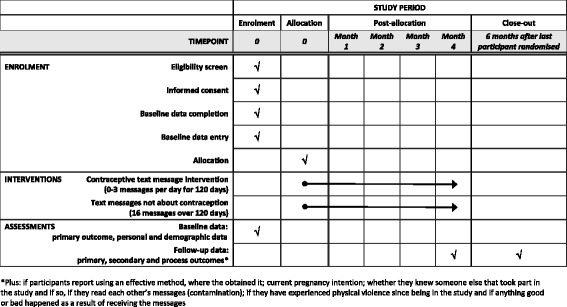
Schedule of enrolment, interventions and assessments. *Plus: if participants report using an effective method, where they obtained it; current pregnancy intention; whether they knew someone else that took part in the study and, if so, if they read each other’s messages (contamination); if they have experienced physical violence since being in the study and if anything good or bad happened as a result of receiving the messages

See Fig. 2 for the schedule of enrolment, interventions and assessments.

### Allocation and protecting against bias

Randomisation will occur immediately after baseline data is submitted on the trial database and randomisation system. Participants will receive the first message the day after they install the app. The allocation sequence is generated by the remote, computer-based randomisation software, ensuring that investigators are unaware of allocation before participants are randomised. Due to the nature of the intervention, participants will be aware of the allocation soon after they start receiving the messages. Local research staff collecting outcome data will not be aware of allocation unless this is revealed to them by the participant. Researchers who analyse the data will be masked to treatment allocation.

### Intervention delivery

After randomisation, the trial database and randomisation system will send the local SMS platform the following information: allocation, time slot (participants can choose to receive messages from 10:00 to 13:59, 14:00 to 18:59, or both), mobile phone number and marital status. The platform will send the intervention or control messages. Messages sent will be recorded by the local platform and will be monitored.

### Sample size

The trial is powered to detect an increase in acceptability of effective contraception. Four hundred and fifty-four participants will allow for 90% power to detect a 15% absolute increase in acceptability, assuming 50% acceptability in the control group (i.e. 50% in the control vs. 65% in the intervention, corresponding to an odds ratio of 1.86). The sample size was calculated using the statistical software Stata 15.0 (syntax: power twoproportions .50 .65, test(chi2) power(0.9)) [68]. A 50% acceptability baseline is used in the absence of published data on acceptability in this context. If the actual baseline acceptability is higher or lower than 50%, the trial is still sufficiently powered to detect an absolute difference of 15%. For example, if the proportion in the control arm is 75%, there will be 90% power to detect an absolute difference of 12% (corresponding to 87% acceptability in the intervention group and an odds ratio of 2.23). Allowing for 20% loss to follow-up, 570 people will be randomised.

### Data management

We did not convene a Data Monitoring and Ethics Committee as the intervention provides support and is unlikely to produce adverse effects. However, we have convened a Trial Steering Committee and they have agreed to take on the monitoring of ethical aspects of the trial. The trial sponsor may audit the trial according to their own risk assessment and schedule.

Personal details entered onto the trial database and randomisation system will be stored on LSHTM’s secure server. Personally identifiable information exported from the database will be stored separately from anonymised research data on an LSHTM computer. Participant mobile phone numbers, but no other personal details, will be stored in the local platform that sends out the text messages. Signed paper Consent Forms and questionnaires will be kept in a data enclave at PFPPA. All data arising from the study will be kept confidential and is only accessible to researchers directly involved in it. Personally identifiable data will not be kept longer than necessary and will be deleted within 3 months following study completion. We will retain primary research data for 10 years following study completion.

### Analyses

#### General statistical considerations

The analysis of the data will follow the plan specified below. There will be no interim analyses and, therefore, no stopping rules. All analyses will be according to randomised arm and only participants with complete outcome data will be included in the primary analysis (a complete case analysis). All statistical tests will be two-sided. All effect estimates will be reported with a 95% confidence interval and its associated *p* value. Statistical significance will be considered at the 5% level. Analyses will be conducted using the latest version of Stata.

#### Loss to follow-up

To investigate whether loss to follow-up differs by arm, we will report this descriptively and use a﻿ chi-squared test. We will use logistic regression to compare baseline characteristics of participants who completed 4-month follow-up against participants who did not. We will report predictors of loss to follow-up and investigate whether the effect of these differs by arm by testing for an interaction.

#### Assumptions about missing data

As we are not aware of similar trials, it is not possible to investigate the pattern of missing data. The complete case analysis assumes that missing data for participants who did not complete follow-up are similar to data from participants who did complete follow-up, conditionally on baseline covariates included in the analysis model (i.e. that data is missing at random (MAR)) [69]. If participants who complete follow-up are more likely to find an effective method acceptable compared to those that are lost to follow-up, the observed proportion may overestimate acceptability [69]. European Union guidance on missing data in clinical trials highlights this limitation of MAR among others [70]. The guidance states, however, that ‘under reasonable assumptions’ the estimate of treatment effect is unlikely to be biased ‘to an important degree’. We will conduct the principal analysis under a MAR assumption (conditionally on the adjustment variables in the model), then perform sensitivity analysis under different assumptions for the missing data, as explained below.

#### Missing covariates

The database requires all items on the baseline questionnaire to be submitted to randomisation. Therefore, there will be no missing baseline covariates.

#### Principal analyses

##### Descriptive analysis

We will report a flow diagram of trial participation, as recommended in the CONSORT guidelines [71]. We will report the baseline characteristics by treatment arm. We will also explore the baseline factors associated with retention (see above).

##### Analysis of the primary outcome

The primary outcome is binary and we will compare the crude proportion reporting that at least one method is acceptable in each group. We will estimate the difference between the groups using logistic regression and will report the odds ratio along with the 95% confidence interval and its *p* value for evidence against the absence of intervention effect from the model. The primary analysis regression will be adjusted for baseline covariates likely to be associated with the outcome in order to improve the efficiency of the analysis and avoid chance imbalances [72]. These pre-specified covariates that we will adjust for are: pregnancy intention (wants to avoid/other); age (18–19/20–24 years); number of children (0/1+); highest education level completed (university/other) and acceptability of effective contraception at baseline (at least one method acceptable/no methods acceptable). We will also report the crude odds ratio between arms.

##### Analysis of the secondary outcomes

The analysis of the secondary outcomes will be the similar to the analysis of the primary outcome. We will estimate the difference between the groups using logistic regression, report odds ratios with 95% confidence intervals and their *p* values. All regressions will be adjusted for the pre-specified covariates as above (although with the acceptability of individual methods, the outcome at baseline will replace acceptability of effective contraception).

##### Analysis of the process outcomes

The process outcomes perceived norms, personal agency and intention are comprised of ordinal scales. Each scale will be analysed individually using ordered logistic regression to estimate proportional odds ratios. For knowledge, each correct answer will receive 1 point. The points will be summed and an overall score will be produced. We will use linear regression to test for a difference in mean scores between the arms.

To assess the ‘dose’ of the intervention that the intervention participants received, we will analyse the number of messages that participants reported to have read (all, most, some, none) and whether they stopped the messages. This will be reported descriptively.

#### Additional analyses

##### Sensitivity analyses

We will conduct sensitivity analyses regarding the missing data assumptions. In the first sensitivity analysis, we will consider that data are not MAR, and that all participants lost to follow-up did not find at least one method acceptable. In the second, we will adjust for the main baseline predictors of missingness. Both sensitivity analyses will be adjusted for the pre-specified covariates as above.

##### Subgroup analysis

Recognising that the trial is not powered to detect effect differences in subgroups, we will conduct exploratory subgroup analyses for the primary outcome to determine if the intervention effect varies by baseline characteristics. The pre-specified subgroups are: age (split at the median); marital status (married/not married); number of children (0/1+); residence (city/other); occupation (in education/other); highest education level completed (university/other) and pregnancy intention (wants to avoid/other). Within the pre-specified subgroups, we will assess heterogeneity of treatment effect with a test for interaction [73–77]. Interaction test *p* values will be presented but will be interpreted with caution due to the exploratory nature, the multiple tests performed and of the low power of the interaction test. We will estimate odds ratios along with 95% CIs for each subgroup without *p* values. As this is an exploratory analysis of potentially influential characteristics that are not justified a priori, we will not hypothesise effect directions.

##### Contamination

To assess the potential for contamination, we will report the proportion of control group participants who read another participant’s messages and the proportion of intervention participants whose messages were read by another participant.

##### Analysis of pooled trial data

We are conducting trials of similar interventions in two other countries. If the results of the other trials are available, we will conduct the principal analyses on the pooled dataset.

### Participants’ rights and safety

Participants will have the right to withdraw at any time during their involvement, without having to give a reason. Participants can withdraw by contacting the project coordinator. Acting on participants’ requests to withdraw from the trial, participants’ status will be changed to ‘withdrawn’, the text messages will stop and the person will be excluded from the list of participants who are due follow-up. Participants will be able to stop text messages, but choose to continue with the trial follow-up. Participants’ participation and personal identifiable data will remain confidential and research data will be anonymised.

In the formative work, we explored young people’s views on confidentiality about receiving messages on their mobile phone. While the large majority of participants did not report that they were concerned about receiving messages about contraception on their mobile phone, it is possible that some participants will want to keep the messages confidential from certain people (e.g. partner, parents) and that these people might view the messages. The messages remind participants that they can delete the messages and provide instructions on how to keep the messages private. We will provide information on support services that they can contact if they feel unsafe because of the messages being read. We will review physical violence during participants’ involvement in the trial reported on the follow-up questionnaire.

## Discussion

The results of this trial will provide evidence for the effect of the intervention on young Palestinian women’s attitudes towards effective contraception. The analysis of the secondary and process outcomes may provide evidence for the effect of the intervention on the use of effective contraception, attitudes towards the individual effective methods, service use, unintended pregnancy, induced abortion and on the psychological processes hypothesised to influence contraceptive use.

There are a number of limitations of this trial. The main limitation is that the primary outcome is self-reported. As sexual activity before marriage is highly stigmatised in Palestine, this precluded the option of an objective primary outcome in the study population. We expect that a significant proportion of participants in the trial will not be married and either will not be sexually active, or will be unwilling to admit if they are. Powering a study for uptake of contraception in a population, which will likely result in a small number of outcome events, would make the sample size prohibitively large. In not married/not sexually active young women, the intervention aims to improve attitudes towards the most effective methods, so that they will find a wider range of methods acceptable if they want to choose a method in the future.

While local research staff are not made aware of allocation, there is a chance that the participants could reveal to local research staff collecting outcome data the group that they were allocated to. Staff are trained in asking questions in a standardised way. As the communities from which the trial participants derive are close, we anticipate that there will be some degree of contamination but we cannot predict the extent of it. We are measuring the potential for contamination at 4-month follow-up and will consider this when interpreting the results. In absence of further data, we have powered the trial to detect an absolute difference of 15% in the proportion of participants finding at least one method of effective contraceptive acceptable. The trial will have a lower power if the intervention has a smaller effect.

### Trial status

Recruitment commenced on 8 December 2016 and is will be complete by 31 July 2017. The estimated completion date for the final participant recruited (final data collection date for the primary outcome) is January 2018.

## Additional files

Additional file 1: Baseline questionnaire. Questionnaire completed after informed consent and before randomisation. (DOCX 16 kb)

Additional file 2: Follow-up questionnaire. Questionnaire completed 4 months after randomisation. (DOCX 20 kb)

Additional file 3: Trial Information Sheet. Participant Trial Information Sheet. (DOCX 18 kb)

Additional file 4: Trial Consent Form. Participant Trial Consent Form. (DOCX 27 kb)

## Abbreviations

CONSORT: Consolidated Standards of Reporting Trials diagram
OC: Oral contraceptive pill
IUD: Intrauterine device
LSHTM: The London School of Hygiene and Tropical Medicine
PFPPA: the Palestinian Family Planning and Protection Association
IPPF: The International Planned Parenthood Federation.
MAR: Missing at random
SMS: Short messaging service
