# Supplementary material for: An intervention delivered by text message to increase the acceptability of effective contraception among young women in Palestine: study protocol for a randomised controlled trial
Source: Trials. 2017 Oct 3;18:454. doi: 10.1186/s13063-017-2191-1 (PMC5627444; doi:10.1186/s13063-017-2191-1)
Supplement: Supplementary file 1 — Baseline questionnaire. Questionnaire completed after informed consent and before randomisation. (DOCX 16 kb) [file 13063_2017_2191_MOESM1_ESM.docx]

Additional file 1. Baseline questionnaire

| **Thank you very much for taking part in the study. Please complete the following questionnaire. Please be as honest as possible. All of your answers will remain confidential.** | | | | | | | | | | | | | | |
| --- | --- | --- | --- | --- | --- | --- | --- | --- | --- | --- | --- | --- | --- | --- |
|  | ***Using the pill…*** | |  | |  | |  | |  | |  | |  | |
| 1 | causes infertility | | Strongly disagree | | Disagree | | Not sure | | Agree | | Strongly agree | | I do not know what the pill is | |
| 2 | causes unwanted side-effects | | Strongly disagree | | Disagree | | Not sure | | Agree | | Strongly agree | | I do not know what the pill is | |
| 3 | is easy | | Strongly disagree | | Disagree | | Not sure | | Agree | | Strongly agree | | I do not know what the pill is | |
| 4 | is a good way to prevent pregnancy | | Strongly disagree | | Disagree | | Not sure | | Agree | | Strongly agree | | I do not know what the pill is | |
| 5 | I would recommend the pill to a friend | | Strongly disagree | | Disagree | | Not sure | | Agree | | Strongly agree | | I do not know what the pill is | |
|  | ***Using the IUD…*** | |  | |  | |  | |  | |  | |  | |
| 6 | causes infertility | | Strongly disagree | | Disagree | | Not sure | | Agree | | Strongly agree | | I do not know what the IUD is | |
| 7 | causes unwanted side-effects | | Strongly disagree | | Disagree | | Not sure | | Agree | | Strongly agree | | I do not know what the IUD is | |
| 8 | is easy | | Strongly disagree | | Disagree | | Not sure | | Agree | | Strongly agree | | I do not know what the IUD is | |
| 9 | is a good way to prevent pregnancy | | Strongly disagree | | Disagree | | Not sure | | Agree | | Strongly agree | | I do not know what the IUD is | |
| 10 | I would recommend the IUD to a friend | | Strongly disagree | | Disagree | | Not sure | | Agree | | Strongly agree | | I do not know what the IUD is | |
| 11 | The IUD insertion would not be a problem | | Strongly disagree | | Disagree | | Not sure | | Agree | | Strongly agree | | I do not know what the IUD is | |
|  | ***Using the injection…*** | |  | |  | |  | |  | |  | |  | |
| 12 | causes infertility | | Strongly disagree | | Disagree | | Not sure | | Agree | | Strongly agree | | I do not know what the injection is | |
| 13 | causes unwanted side-effects | | Strongly disagree | | Disagree | | Not sure | | Agree | | Strongly agree | | I do not know what the injection is | |
| 14 | is easy | | Strongly disagree | | Disagree | | Not sure | | Agree | | Strongly agree | | I do not know what the injection is | |
| 15 | is a good way to prevent pregnancy | | Strongly disagree | | Disagree | | Not sure | | Agree | | Strongly agree | | I do not know what the injection is | |
| 16 | I would recommend the injection to a friend | | Strongly disagree | | Disagree | | Not sure | | Agree | | Strongly agree | | I do not know what the injection is | |
|  | ***Using the implant…*** | |  | |  | |  | |  | |  | |  | |
| 17 | causes infertility | | Strongly disagree | | Disagree | | Not sure | | Agree | | Strongly agree | | I do not know what the implant is | |
| 18 | causes unwanted side-effects | | Strongly disagree | | Disagree | | Not sure | | Agree | | Strongly agree | | I do not know what the implant is | |
| 19 | is easy | | Strongly disagree | | Disagree | | Not sure | | Agree | | Strongly agree | | I do not know what the implant is | |
| 20 | is a good way to prevent pregnancy | | Strongly disagree | | Disagree | | Not sure | | Agree | | Strongly agree | | I do not know what the implant is | |
| 21 | I would recommend the implant to a friend | | Strongly disagree | | Disagree | | Not sure | | Agree | | Strongly agree | | I do not know what the implant is | |
| 22 | The implant insertion would not be a problem | | Strongly disagree | | Disagree | | Not sure | | Agree | | Strongly agree | | I do not know what the implant is | |
|  | ***Using the patch…*** | |  | |  | |  | |  | |  | |  | |
| 23 | causes infertility | | Strongly disagree | | Disagree | | Not sure | | Agree | | Strongly agree | | I do not know what the patch is | |
| 24 | causes unwanted side-effects | | Strongly disagree | | Disagree | | Not sure | | Agree | | Strongly agree | | I do not know what the patch is | |
| 25 | is easy | | Strongly disagree | | Disagree | | Not sure | | Agree | | Strongly agree | | I do not know what the patch is | |
| 26 | is a good way to prevent pregnancy | | Strongly disagree | | Disagree | | Not sure | | Agree | | Strongly agree | | I do not know what the patch is | |
| 27 | I would recommend the patch to a friend | | Strongly disagree | | Disagree | | Not sure | | Agree | | Strongly agree | | I do not know what the patch is | |
|  |  | |  | |  | |  | |  | |  | |  | |
|  |  | |  | |  | |  | |  | |  | |  | |
| 28 | What is your first name? |  | | | | | | | | | | | | |
| 29 | What is your last name? |  | | | | | | | | | | | | |
| 30 | What is your mobile number? |  | | | | | | | | | | | | |
| 31 | What is your email address? |  | | | | | | | | | | | | |
| 32 | What day were you born? | Day | | Month | | Year | |  | |  | |  | | |
| 33 | Are you? | Married | | Not married | |  | |  | |  | |  | | |
| 34 | How many children do you have? | 0 | | 1 | | 2 or more | |  | |  | |  | | |
| 35 | Where do you live? | City | | Village | | Camp | | Bedouin | |  | |  | | |
| 36 | Are you? (check all that apply) | At school | | At university | | Working | | Training | |  | |  | | |
|  |  | Full time parent | | Not working | | Long-term sick | |  | |  | |  | | |
| 37 | What is the highest level of education that you have completed? | Primary | | Secondary | | University | | Technical education | |  | |  | | |
| 38 | Do you want a pregnancy now? | Yes | | No | | Unsure | | Not married | |  | |  | | |
| 39 | What method of contraception are you using now (check all that apply)? | None | | Injection | | Male condom | | IUD | | Not married | |  | | |
|  |  | Implant | | Pill | | Female condom | | Calendar-based method | | LAM | |  | | |
|  |  | Withdrawal | | Patch | | Ring | | Other method | |  | |  | | |
| 40 | How did you hear about this study? | Facebook | | PFPPA service delivery point | | PFPPA  website | | Friend/family | | Flyer/poster | | Other | | |
| 41 | How did you enrol in this study? | PFPPA Jerusalem | | PFPPA Bethlehem | | PFPPA Halhoul | | PFPPA Hebron | | PFPPA Ramallah | | Youth friendly service | | Online |
| 42 | What times do you prefer to receive messages? | 10-2 | | 2-6 | | 10-6 | |  | |  | |  | | |
