# Supplementary material for: An intervention delivered by text message to increase the acceptability of effective contraception among young women in Palestine: study protocol for a randomised controlled trial
Source: Trials. 2017 Oct 3;18:454. doi: 10.1186/s13063-017-2191-1 (PMC5627444; doi:10.1186/s13063-017-2191-1)
Supplement: Supplementary file 2 — Follow-up questionnaire. Questionnaire completed 4 months after randomisation. (DOCX 20 kb) [file 13063_2017_2191_MOESM2_ESM.docx]

Additional file 2. Follow-up questionnaire

| **Thank you very much for taking part in the study. Now it is time to complete the final questionnaire. Please be as honest as possible. All of your answers will remain confidential.** | | | | | | | | | | | |
| --- | --- | --- | --- | --- | --- | --- | --- | --- | --- | --- | --- |
| 1 | What method of contraception are you using now (check all that apply)? | None | | Injection | | Male condom | | IUD | | | Not married |
|  |  | Implant | | Pill | | Female condom | | Calendar-based method | | | LAM |
|  |  | Withdrawal | | Patch | | Ring | | Other method | | |  |
| 2 | Where did you get this method? | PFPPA Jerusalem | | PFPPA  Bethlehem | | PFPPA  Halhoul | | PFPPA  Hebron | | | PFPPA Ramallah |
|  |  | MoH clinic | | UNRWA | | Not using a method | | I do not know | | | Not married |
| 3 | Do you want a pregnancy now? | Yes | | No | | Not sure | | Not married | | |  |
|  |  |  |  |  | |  |  | | |  | |
|  | ***Using the pill…*** |  |  |  | |  |  | | |  | |
| 4 | causes infertility | Strongly disagree | Disagree | Not sure | | Agree | Strongly agree | | | I do not know what the pill is | |
| 5 | causes unwanted side-effects | Strongly disagree | Disagree | Not sure | | Agree | Strongly agree | | | I do not know what the pill is | |
| 6 | is easy | Strongly disagree | Disagree | Not sure | | Agree | Strongly agree | | | I do not know what the pill is | |
| 7 | is a good way to prevent pregnancy | Strongly disagree | Disagree | Not sure | | Agree | Strongly agree | | | I do not know what the pill is | |
| 8 | I would recommend the pill to a friend | Strongly disagree | Disagree | Not sure | | Agree | Strongly agree | | | I do not know what the pill is | |
|  | ***Using the IUD…*** |  |  |  | |  |  | | |  | |
| 9 | causes infertility | Strongly disagree | Disagree | Not sure | | Agree | Strongly agree | | | I do not know what the IUD is | |
| 10 | causes unwanted side-effects | Strongly disagree | Disagree | Not sure | | Agree | Strongly agree | | | I do not know what the IUD is | |
| 11 | is easy | Strongly disagree | Disagree | Not sure | | Agree | Strongly agree | | | I do not know what the IUD is | |
| 12 | is a good way to prevent pregnancy | Strongly disagree | Disagree | Not sure | | Agree | Strongly agree | | | I do not know what the IUD is | |
| 13 | I would recommend the IUD to a friend | Strongly disagree | Disagree | Not sure | | Agree | Strongly agree | | | I do not know what the IUD is | |
| 14 | The IUD insertion would not be a problem | Strongly disagree | Disagree | Not sure | | Agree | Strongly agree | | | I do not know what the IUD is | |
|  | ***Using the injection…*** |  |  |  | |  |  | | |  | |
| 15 | causes infertility | Strongly disagree | Disagree | Not sure | | Agree | Strongly agree | | | I do not know what the injection is | |
| 16 | causes unwanted side-effects | Strongly disagree | Disagree | Not sure | | Agree | Strongly agree | | | I do not know what the injection is | |
| 17 | is easy | Strongly disagree | Disagree | Not sure | | Agree | Strongly agree | | | I do not know what the injection is | |
| 18 | is a good way to prevent pregnancy | Strongly disagree | Disagree | Not sure | | Agree | Strongly agree | | | I do not know what the injection is | |
| 19 | I would recommend the injection to a friend | Strongly disagree | Disagree | Not sure | | Agree | Strongly agree | | | I do not know what the injection is | |
|  | ***Using the implant…*** |  |  |  | |  |  | | |  | |
| 20 | causes infertility | Strongly disagree | Disagree | Not sure | | Agree | Strongly agree | | | I do not know what the implant is | |
| 21 | causes unwanted side-effects | Strongly disagree | Disagree | Not sure | | Agree | Strongly agree | | | I do not know what the implant is | |
| 22 | is easy | Strongly disagree | Disagree | Not sure | | Agree | Strongly agree | | | I do not know what the implant is | |
| 23 | is a good way to prevent pregnancy | Strongly disagree | Disagree | Not sure | | Agree | Strongly agree | | | I do not know what the implant is | |
| 24 | I would recommend the implant to a friend | Strongly disagree | Disagree | Not sure | | Agree | Strongly agree | | | I do not know what the implant is | |
| 25 | The implant insertion would not be a problem | Strongly disagree | Disagree | Not sure | | Agree | Strongly agree | | | I do not know what the implant is | |
|  | ***Using the patch…*** |  |  |  | |  |  | | |  | |
| 26 | causes infertility | Strongly disagree | Disagree | Not sure | | Agree | Strongly agree | | | I do not know what the patch is | |
| 27 | causes unwanted side-effects | Strongly disagree | Disagree | Not sure | | Agree | Strongly agree | | | I do not know what the patch is | |
| 28 | is easy | Strongly disagree | Disagree | Not sure | | Agree | Strongly agree | | | I do not know what the patch is | |
| 29 | is a good way to prevent pregnancy | Strongly disagree | Disagree | Not sure | | Agree | Strongly agree | | | I do not know what the patch is | |
| 30 | I would recommend the patch to a friend | Strongly disagree | Disagree | Not sure | | Agree | Strongly agree | | | I do not know what the patch is | |
|  |  |  | |  | |  | |  | | |  |
|  |  |  | |  | |  | |  | | |  |
| 31 | Have you become pregnant since joining this study? | Yes | | No | | Not married | |  | | |  |
| 32 | Did you want to become pregnant during this study? | Yes | | No | | Not sure | | Not married | | |  |
| 33 | Have you had a termination (abortion) since joining this study? | Yes | | No | | Not married | |  | | |  |
| 34 | What method of contraception have you used during this study (check all that apply)? | None | | Injection | | Male condom | | IUD | | | Not married |
|  |  | Implant | | Pill | | Female condom | | Calendar-based method | | | LAM |
|  |  | Withdrawal | | Patch | | Ring | | Other method | | |  |
| 35 | How many times have you attended a sexual health service since you joined this study 4 months ago? | 0 | | 1 | | 2 or more | |  | | |  |
| 36 | How many messages did you read? | All | | Most | | Some | | None | | |  |
| 37 | Did you stop the messages? | Yes | | No | |  | |  | | |  |
| 38 | Do you know anyone else in the study? | Yes | | No | |  | |  | | |  |
| 39 | Did they read the messages that we sent you? | Yes | | No | | I do not know anyone in the study | | | | |  |
| 40 | Did you read the messages that we sent them? | Yes | | No | | I do not know anyone in the study | | | | |  |
| 41 | Have you experienced physical violence since being in the study? | Yes | | No | |  | |  | | |  |
| 42 | Did anything good or bad happen as a result of receiving the messages? If so, please summarise here: |  | | | | | | | | | |
|  |  |  | | | | | | | | | |
| 43 | Hormonal contraception is more effective at preventing pregnancy than condoms alone | Disagree | | | Agree | | | | Do not know | | |
| 44 | The pill is taken once a month | Disagree | | | Agree | | | | Do not know | | |
| 45 | The IUD lasts for 6 months | Disagree | | | Agree | | | | Do not know | | |
| 46 | The injection is given once a year | Disagree | | | Agree | | | | Do not know | | |
| 47 | The implant can stay under the skin for 10 years | Disagree | | | Agree | | | | Do not know | | |
| 48 | A new patch is worn each week for 3 weeks, then no patch for the 4^th^ week | Disagree | | | Agree | | | | Do not know | | |
| 49 | I know where to get contraception | Disagree | | | Agree | | | | Do not know | | |

| **If you are not married, please imagine that you are and try to answer the following questions:** | | | | | | |
| --- | --- | --- | --- | --- | --- | --- |
| 50 | My friends would use the pill, IUD, injection, implant or patch if they wanted to prevent pregnancy. | Strongly disagree | Disagree | Not sure | Agree | Strongly agree |
| 51 | My friends would talk to their husband about contraception if they wanted to prevent a pregnancy. | Strongly disagree | Disagree | Not sure | Agree | Strongly agree |
|  |  |  |  |  |  |  |
| 52 | If you wanted to use the pill, IUD, injection, implant or patch, how easy would it be for you to use it? | Very difficult | Difficult | Not sure | Easy | Very easy |
| 53 | If you wanted to talk to your husband about contraception, how easy would it be for you to talk to him? | Very difficult | Difficult | Not sure | Easy | Very easy |
|  |  |  |  |  |  |  |
| 54 | If you wanted to use the pill, IUD, injection, implant or patch, how certain are you that you could use it? | Very certain I could not | Certain I could not | Not sure | Certain I could | Very certain I could |
| 55 | If you wanted to talk to your husband about contraception, how certain are you that you could talk to him? | Very certain I could not | Certain I could not | Not sure | Certain I could | Very certain I could |
|  |  |  |  |  |  |  |
| 56 | I intend to use the pill, IUD, injection, implant or patch | Strongly disagree | Disagree | Not sure | Agree | Strongly agree |
