# Supplementary material for: An intervention delivered by text message to increase the acceptability of effective contraception among young women in Palestine: study protocol for a randomised controlled trial
Source: Trials. 2017 Oct 3;18:454. doi: 10.1186/s13063-017-2191-1 (PMC5627444; doi:10.1186/s13063-017-2191-1)
Supplement: Supplementary file 3 — Trial Information Sheet. Participant Trial Information Sheet. (DOCX 18 kb) [file 13063_2017_2191_MOESM3_ESM.docx]

Additional file 3. Trial information sheet

**We are inviting you to take part in a research study. Before you decide, it is important that you know why we are doing the study and what is involved. Please read the following information carefully.**

#### What is the study?

The study is testing whether SMS messages providing information and support helps change young women’s attitudes towards the most effective methods of contraception.

#### Who is organising and funding the study?

The study is being conducted by the Palestinian Family Planning and Protection Association (PFPPA) and the London School of Hygiene & Tropical Medicine (LSHTM). PFPPA provides high quality sexual and reproductive health services in the Palestinian Authority. LSHTM is a world-leading centre for research and postgraduate education in public and global health. The study is funded by the International Planned Parenthood Federation (IPPF).

#### Why have I been chosen?

You are female, aged 18-24, do not report using effective contraception, own a personal mobile phone and live in the West Bank.

#### Do I have to take part?

No, you do not have to take part. It is your choice. If you choose not to take part, all the services you receive will continue as normal.

#### What will happen if I take part?

After you have had all of your questions answered and if you decide to take part, we will ask you to complete a consent form. We will then ask you to complete a confidential questionnaire that asks you to provide details about yourself, such as name and date of birth. The questionnaire also asks for your feelings and attitudes towards contraception. Completing the questionnaire will take 5-10 minutes. You can complete the consent and questionnaire on our secure and confidential study website or by filling out a paper version.

After you have completed the questionnaire, **an automated computer system will put you into one of two groups by chance (randomly)**:

Group 1: One group will receive 0 to 3 text messages a day about contraception over 4 months.

Group 2: The other group will receive SMS messages that are not about contraception. This group will receive one message a month for 4 months.

At the end of the study, 4 months after joining, we will ask you to complete the questionnaire again. This is to see how things may have changed. You can complete the 4-month questionnaire on our secure and confidential study website, by filling out a paper version at the service or by providing your answers to the research staff over the phone.

#### Will you compensate me for taking part?

Unfortunately, we are not able to offer you something for taking part. You do not have to pay for the messages that you receive.

#### What are the alternatives?

You do not have to take part.

#### What are the possible disadvantages of taking part?

Completing the questionnaires will take some of your time. It is possible that the messages that we send could be read by someone else. If you are concerned about this, you could lock your phone and delete the messages after you read them. You can choose the times that you want to receive the messages.

#### What are the possible advantages of taking part?

You may find the messages helpful and enjoy the experience of taking part in research. You can add your participation in the study to your resume. You can add your participation in the study to your resume.

#### What if I do not want to take part anymore?

You can stop receiving messages by texting ‘stop’. You can leave the study at any time by contacting the Project Coordinator (details below). You do not have to give a reason for wanting to leave the study. Leaving the study will not affect the services that you receive.

#### What if there is a problem?

You can talk to the Project Coordinator at any time (details below).

#### Will my taking part be confidential?

Yes. Your answers to the questionnaires will be stored anonymously and your contact details and will be kept confidential and separate from your answers to the questionnaires. You will be assigned a unique study number when you join the study. We will not inform your parents, partner or anyone else about your involvement in this research. With your permission, will search PFPPA records to check for any services you may have received during your time in the study.

#### What will happen with the results of this study?

We will share the results through publication in journals and through conference presentations. If you would like to know the results of the study, please contact the team (details below) and we will share them with you. Your name will not be used in the results of this study.

If the results of the study show that the messages have helped, they will be made available to all young people in the West Bank.

#### Who has reviewed this study?

The London School of Hygiene & Tropical Medicine Interventions Research Ethics Committee and the State of Palestine Ministry of Health.

**Thank you for taking the time to consider taking part**

| **Sarah Abu Ghazaleh** | **Amina Stavridis** | **Ona McCarthy** |
| --- | --- | --- |
| Administrative Assistant | Executive Director | Principal Investigator |
| PFPPA, Industrial Zone, Wadi Al-Joze | PFPPA, Industrial Zone, Wadi Al-Joze | LSHTM, Keppel St, WC1E 7HT |
| Jerusalem, Palestinian Territory | Jerusalem, Palestinian Territory | London, United Kingdom |
| [ipc@pfppa.org](mailto:ipc@pfppa.org) | [director@pfppa.org](mailto:director@pfppa.org) | [ona.mccarthy@lshtm.ac.uk](mailto:ona.mccarthy@lshtm.ac.uk) |
| [+ 9722-6280630](tel:+97226280630) |  |  |
